# 1427. Healthcare Resource Utilization During Hospitalizations with UTI in the US, 2018

**DOI:** 10.1093/ofid/ofab466.1619

**Published:** 2021-12-04

**Authors:** Marya Zilberberg, Brian Nathanson, Kate Sulham

**Affiliations:** 1 EviMed Research Group, LLC, Goshen, MA; 2 OptiStatim, LLC, Longmeadow, MA; 3 Spero Therapeutics, Cambridge, MA

## Abstract

**Background:**

Urinary tract infection (UTI) as the reason for hospitalization costs the US healthcare system nearly &3 billion annually, and is on the rise. We set out to explore the full burden of UTI hospitalizations in the US, including admissions both *for* UTI and *with* UTI.

**Methods:**

We conducted a cross-sectional multicenter study within the National Inpatient Sample (NIS) database, a 20% stratified sample of discharges from US hospitals, from 2018, to explore hospital resource utilization of patients discharged with a UTI diagnosis. We divided UTI into mutually exclusive categories of complicated (cUTI), uncomplicated (uUTI), and catheter-associated (CAUTI), in addition to healthcare-associated (HAUTI). We calculated unadjusted hospital charges, costs, average reimbursements, and length of stay (LOS) associated with these infections.

**Results:**

Among 2,837,385 discharges with a UTI code, 77.9% were uUTI, 17.6% cUTI (80.2% HAUTI), and 4.4% CAUTI; UTI was principal diagnosis in only 17.0%. Median [interquartile range, IQR] LOS ranged from 4 [3-8] days in uUTI and cUTI to 5 [3-9] days in CAUTI. Overall median [IQR] hospital charges and costs were lowest in uUTI (&36,335 [&19,920-&70,745] and &8,898 [&5,408-&16,092], respectively) and highest in cUTI (&39,690 [&21,997-&75,739] and &9,713 [&5,923-&17,423], respectively), with the HAUTI subgroup being most costly (&44,650 [&24,642-&85,628] and &10,945 [&6,573-&19,634], respectively). “Septicemia or Severe Sepsis without MV >96 Hours with MCC” was the most common DRG in uUTI (13.2%) and cUTI (14.2%), with the corresponding median [IQR] reimbursements of &11,057 [&7,028-&17,757] and &12,226 [&7,889-&19,216], respectively. In contrast, CAUTI was most commonly (44.7%) reimbursed under “Kidney and Urinary Tract Infections without MCC” at &8,635 [&5,693-&13,718].

**Conclusion:**

The nearly 3 million hospital admissions with a UTI represent 8% of all annual admissions in the US. Though the majority are considered uncomplicated, all categories are nearly equally costly. Given that over 80% of all UTI-associated admissions are with UTI as a secondary diagnosis, annual estimates of primary UTI costs likely significantly underrepresent the true economic burden of UTI on the US healthcare system.

**Disclosures:**

**Marya Zilberberg, MD, MPH**, **Cleveland Clinic** (Consultant)**J&J** (Shareholder)**Lungpacer** (Consultant, Grant/Research Support)**Merck** (Grant/Research Support)**scPharma** (Consultant)**Sedana** (Consultant, Grant/Research Support)**Spero** (Grant/Research Support) **Brian Nathanson, PhD**, **Lungpacer** (Grant/Research Support)**Merck** (Grant/Research Support)**Spero** (Grant/Research Support) **Kate Sulham, MPH**, **Spero Therapeutics** (Consultant)

